# Supplementary material for: Synthetic data distillation enables the extraction of clinical information at scale
Source: NPJ Digit Med. 2025 May 10;8:267. doi: 10.1038/s41746-025-01681-4 (PMC12065832; doi:10.1038/s41746-025-01681-4)
Supplement: Supplementary file 1 — Supplementary Information [file 41746_2025_1681_MOESM1_ESM.pdf]

Supplementary Information

Supplementary Table 1. Synthetic data format and annotation summary
We manually annotated 1000 fine-tuning examples generated by Llama-3.1-70B. Overall, 872 of the questions remained unchanged after manual review. A breakdown by question type occurs in the following table. The column “occurrence in data” corresponds to the frequency with which the question occurred in the dataset and the “percentage requiring editing” corresponds to how often that type of question needed to be edited. The “request provided” and “example provided” both correspond to sections in the prompt used during data generation.

Type	Occurrence in data	Percentage requiring editing	Request provided	Example provided
boolean	29.1%	15.6%	Give a list of 10 total, different, patient note-specific questions similar to clinical trial eligibility criteria. 5 should have 'No' as the correct answer and 5 should have 'Yes' as the answer.	```json<br>[<br>{{<br>"question" : "Does the note state that the patient is breathing normally on room air?",<br>"type": "Yes/No",<br>"answer": "No",<br>"section": "History of Present Illness",<br>"difficulty": "2",<br>"source": "She currently is dependent on oxygen and wears 1.5-2 liters around the clock",<br>"explanation": "The note states that she relies on oxygen and provides the amount as 1.5-2 liters so she is not breathing room air. We can assume since she is receiving o2 supplementation and dependent on it, she cannot breathe normally on room air."<br>}},<br>]```
numeric	23.6%	5.8%	Give a list of ten different, realistic, patient note-specific questions similar to clinical trial eligibility criteria with a numeric answer (only generate questions if numeric answers are appropriate, otherwise end the response). All questions should be specific, many numeric values can be listed more than once so make sure to specify first, last, at admission, on discharge, highest, lowest, on a specific date, within ED / ICU etc..	```json<br>[<br>{{<br>"question": "What was the patient's highest creatinine measurement recorded in the note?",<br>"type": "Numeric",<br>"answer": "1.4",<br>"section": "Pertinent Results",<br>"difficulty": "4",<br>"source": "12/03/2023: CREAT: 1.4 \n 12/07/2023: CREAT: 1.1",<br>"explanation": "The highest CREAT measurement was 1.4 because the only other creatinine measurement was 1.1 on 12/07/2023."<br>}},<br>]```

na-boolean	24.1%	14.8%	Give a list of 5 Yes/No questions that are not answerable using the note. These should be questions which seem like they would be applicable to this patient and are similar to clinical trial eligibility criteria but cannot be answered based on the information in the note. These questions need to be things where the answer cannot be assumed simply because something is not mentioned (e.g., They should not be questions about whether the patient has been diagnosed with serious or chronic diseases because if they were it would be mentioned in the note, since it is not mentioned we can assume the answer is no rather than NA. Do not generate questions where Yes or No is known or can be inferred.	```json<br>[<br>{{<br>"question" : "Does the note state the patient has ever taken aspirin for MI prevention?",<br>"type": "Yes/No",<br>"answer" : "N/A",<br>"section": "Not Found",<br>"source" : "Not in Note",<br>"difficulty": "4",<br>"explanation": "The note does not include medication history. It only includes medications prescribed during this encounter. If there were a medication history we would check the list to see if is present. If it was present we would answer Yes, if it were not present we would answer No but because there is no medication history we answer N/A"<br>}},<br>]```
na-numeric	23.2%	13.4%	Give a list of 5 questions asking for numeric answers but where the note does not contain the answer. These should be questions which seem like they would be applicable to this patient and are similar to clinical trial eligibility criteria but cannot be answered based on the information in the note.	```json<br>[<br>{{<br>"question" : "What was the patient's highest A1C recorded in the note during the hospitalization?",<br>"type": "Yes/No",<br>"answer" : "N/A",<br>"section": "Not Found",<br>"source" : "Not in Note",<br>"difficulty": "4",<br>"explanation": "The note does not include an A1C value during the hospitalization and we cannot infer a value for this patient."<br>}},<br>]```

Supplementary Table 2. Number of questions Llama 3.1 70B assigned each difficulty to during the synthetic data generation process.

Difficulty	Boolean	Numeric	NA - Boolean	NA - Numeric
0	0	4	0	0
1	95,963	14,968	223	303
2	89,754	69,031	2,794	3,655
3	22,327	78,494	11,508	12,537
4	3,519	41,809	20,491	17,930
5	416	4,663	32,053	28,579
6	123	530	24,448	22,430

7	9	20	10,116	12,040
8	13	50	3,984	7,029
9	0	1	671	1,733
10	8	71	0	9

Supplementary Table 3. Performance on i2b2 2018 Clinical Trial Eligibility Challenge (Table form of Figure 2). In each row, we take a model fine-tuned on synthetic MIMIC data and evaluate its performance on either the training or test fold from the i2b2 challenge. Parameters are as described in Methods subsection 5.

	Data	Parameters		Balanced Accuracy	Micro-F1
		Temperature	Top_p		
8B	Train	0	1	0.690	0.842
		1	0.5	0.681	0.810
	Test	0	1	0.735	0.847
		1	0.5	0.737	0.819
70B	Train	0	1	0.814	0.901
		1	0.5	0.815	0.897
	Test	0	1	0.840	0.886
		1	0.5	0.835	0.881
Fine-tuned 8B-H-25k	Train	0	1	0.737	0.872
		1	0.5	0.720	0.864
	Test	0	1	0.756	0.875
		1	0.5	0.750	0.881
Fine-tuned	Train	0	1	0.740	0.880

8B-All		1	0.5	0.720	0.883
	Test	0	1	0.760	0.874
		1	0.5	0.745	0.878
Fine-tuned 8B-NB-Only	Train	0	1	0.681	0.828
		1	0.5	0.680	0.832
	Test	0	1	0.703	0.836
		1	0.5	0.680	0.832
Fine-tuned 8B-No-S	Train	0	1	0.632	0.831
		1	0.5	0.626	0.825
	Test	0	1	0.683	0.809
		1	0.5	0.674	0.800

Supplementary Table 4. Apixaban annotated data summary
There were 23 questions (15 boolean, 8 numeric) answered per patient, so for a total of 100 patients there were 2300 questions. Since there are 100 patients, the count of each answer for each question is the same number as the percentage.

Boolean

	Question	Answer	Count (%)
1	Does the note describe the patient as having atrial fibrillation (afib)? Answer "No" if the note describes the patient as having afib secondary to another reversible cause.	Yes	71 (71%)
		No	29 (29%)
2	Does the note describe the patient as ever being diagnosed with depression or major depressive disorder (MDD)? Answer "No" unless the note describes a diagnosis or history of depression.	Yes	23 (23%)
		No	77 (77%)
3	Does the note describe the patient as ever being diagnosed with schizophrenia or any schizoaffective disorders? Answer "No" unless the note describes a	Yes	2 (2%)
		No	98 (98%)

	diagnosis or history of a schizoaffective disorder.		
4	Does the note describe the patient as ever being diagnosed with bipolar disorder? Answer "No" unless the note describes a diagnosis or history of bipolar disorder.	Yes	5 (5%)
		No	95 (95%)
5	Does the note describe the patient as ever having any hemorrhagic tendencies or blood dyscrasias? Answer "No" unless the note describes a diagnosis or history of hemorrhagic tendencies or blood dyscrasias.	Yes	18 (18%)
		No	82 (82%)
6	Does the note describe the patient as having a stroke during this admission or within the last month? (Answer "Yes" for any recent stroke if the date is unclear, answer "No" if no stroke is mentioned or a prior stroke occurred but it was not recent)	Yes	16 (16%)
		No	84 (84%)
7	Does the note describe the patient as ever having peptic ulcer disease?	Yes	6 (6%)
		No	94 (94%)
8	Does the note describe the patient as having serious bleeding in the past 6 months? Answer "No" unless the note describes a serious recent bleeding issue.	Yes	20 (20%)
		No	80 (80%)
9	Does the note describe the patient as having a planned or past ablation procedure for afib? Answer "No" unless the note includes information about a past or planned ablation for afib.	Yes	5 (5%)
		No	95 (95%)
10	Does the note describe the patient as ever having valvular disease (stenosis) requiring surgery? Answer "No" if there is mention of stenosis without surgery.	Yes	10 (10%)
		No	90 (90%)
11	Does the note describe the patient as having heart failure?	Yes	53 (53%)
		No	47 (47%)
12	Does the note describe the patient as having diabetes mellitus (DM1, DM2, T2D, T1DM, T2DM)?	Yes	44 (44%)
		No	56 (56%)
13	Does the note describe the patient as having arterial hypertension (high bp e.g. >140, or HTN)? This includes pre-existing hypertension and treated hypertension.	Yes	82 (82%)
		No	47 (47%)
14	Does the note describe the patient as ever having a stroke or transient ischemic attack (TIA)? Answer "No" unless the note includes information about the patient having a prior stroke or TIA	Yes	19 (19%)
		No	81 (81%)

15	Does the note describe the patient as being unable to make medical decisions upon discharge? Answer "No" unless there is evidence the patient cannot make their own medical decisions. Answer "Yes" if there is clear mention of dementia or the patient is deceased.	Yes	13 (13%)
		No	87 (87%)

Numeric

	Question	Mean value	Median value	Standard deviation	Range	NAs
1	What is the lowest platelet count (PLT) mentioned in the note? Answer "NA" if no platelet count (PLT) is available in the note.	148.53	147.50	90.8	15-364	60 (60%)
2	What is the highest total bilirubin (TotBili, Bili) mentioned in the note? Answer "NA" if no bilirubin value is available in the note.	0.903	0.600	1.11	0.2-6.8	33 (33%)
3	What is the highest aspartate aminotransferase level (AST) mentioned in the note? Answer "NA" if no AST value is available in the note.	194.4	36.0	1049.597	8-8627	33 (33%)
4	What is the highest serum creatinine (Creat) mentioned in the note? Answer "NA" if no creatinine value is available in the note.	1.586	1.200	1.199	0.5-7.8	3 (3%)
5	What is the lowest hemoglobin (HGB) mentioned in the note? Answer "NA" if no HGB value is available in the note.	10.21	10.15	2.054	6.0-15.9	2 (2%)
6	What is the highest CHADS2 score mentioned? Answer "NA" if no CHADS2 score is in	3.95	3.50	1.39	1-6	80 (80%)

	the note.					
7	What is the lowest left ventricular ejection (LVEF, ef, ejection fraction) fraction mentioned in the note? Answer "NA" if no LVEF is in the note, Answer 55 if the lowest value is 55%% or greater.	47.89	50.00	14.4	20-75	53 (53%)
8	What is the highest blood glucose lab mentioned? Answer "NA" if no blood glucose score is in the note.	142.1	126.0	52.2	78-412	3 (3%)

Supplementary Table 5. Hourly GPU rates
This table contains posted hourly rates for 8 x A100 GPU instances in the Eastern US region, rounded to the nearest cent from 3 major providers. The exact hardware specification is found in the Instance column. Websites were available as of September 27, 2024, and are available on the Internet Archive.

Service	Provider	Instance	Price / hour	Source
Azure	Microsoft	ND96asr A100 v4	$27.20	https://azure.microsoft.com/en-us/pricing/details/machine-learning/
cloudML	Google	a2-highgpu-8g	$29.39	https://cloud.google.com/compute/all-pricing
AWS	Amazon	p4d.24xlarge	$32.77	https://aws.amazon.com/ec2/instance-types/p4/

Supplementary Table 6. Example generated questions by type.
The following table contains 3 example questions for each question type.

Type	Question
boolean	Does the patient have a history of septic thrombophlebitis?
	Was the patient's oxygen saturation below 90% upon admission?
	Is the patient's hemoglobin level within the normal range?
numeric	What was the patient's age at admission?
	What was the patient's highest recorded WBC count?
	What was the patient's INR on 2181-5-21?

na-boolean	Is the patient's anemia related to a chronic disease?
	Is the patient a current smoker?
	Has the patient undergone any prior surgery on the right upper extremity?
na-numeric	What is the patient's peak oxygen consumption during a cardiopulmonary exercise test?
	What is the patient's estimated glomerular filtration rate (eGFR) upon admission?
	What is the patient's 6-minute walk test distance?
